# *Vasa*, *Piwi*, and *Pl10* Expression during Sexual Maturation and Asexual Reproduction in the Annelid *Pristina longiseta*

**DOI:** 10.3390/jdb11030034

**Published:** 2023-08-09

**Authors:** Roman P. Kostyuchenko, Natalia P. Smirnova

**Affiliations:** 1 Department of Embryology, St. Petersburg State University, Universitetskaya nab. 7-9, 199034 St. Petersburg, Russia; nataly.paul.smirnova@gmail.com; 2 Unit for Cell Signaling, Department of Immunology and Transfusion Medicine, Oslo University Hospital, 0317 Oslo, Norway; 3 Hybrid Technology Hub-Centre for Organ on a Chip-Technology, Institute of Basic Medical Sciences, University of Oslo, 0317 Oslo, Norway

**Keywords:** *Piwi*, *Vasa*, *Pl10*, stem cells, posterior growth zone, nervous system, fission, asexual reproduction, germline, gametes, gonad development, sexual maturation, *Pristina longiseta*, Naididae, Annelida

## Abstract

Naidids are tiny, transparent freshwater oligochaetes, which are well known for their ability to propagate asexually. Despite the fact that sexually mature individuals and cocoons with embryos are sometimes found in nature, in long-period laboratory cultures, worms reproduce agametically only. In this paper, we showed, for the first time, the expression of *Vasa*, *Piwi*, and *Pl10* homologs in mature *Pristina longiseta* worms with well-developed reproductive system structures and germ cells. Although the animals have been propagated asexually by paratomic fission for over 20 years in our lab, some individuals become sexualized under standard conditions for our laboratory culture and demonstrate various stages of maturation. The fully matured animals developed a complete set of sexual apparatus including spermatheca, atrium, seminal vesicles, and ovisac. They also had a clitellum and were able to form cocoons. The cues for the initiation of sexual maturation are still unknown for *P. longiseta*; nevertheless, our data suggest that the laboratory strain of *P. longiseta* maintains the ability to become fully sexually mature and to establish germline products even after a long period of agametic reproduction. On the other hand, many of the sexualized worms formed a fission zone and continued to reproduce asexually. Thus, in this species, the processes of asexual reproduction and sexual maturation do not preclude each other, and *Vasa*, *Piwi*, and *Pl10* homologs are expressed in both somatic and germline tissue including the posterior growth zone, fission zone, nervous system, germline cells, and gametes.

## 1. Introduction

Reproduction of metazoans occurs mainly by the sexual mode. It is carried out by special types of cells, namely germ cells, or gametes, which are most often formed in permanent or temporary gonads [1,2]. In case of hermaphroditism, the same animal develops both types of germ cells, the male and the female ones. In addition to conspicuous germline cells, the somatic part of the reproductive system may have a complex structure, to ensure both cross-fertilization and embryonic development. Thus, during sexual reproduction, being inherent in virtually all animals, each individual develops various organs necessary for the formation of germ cells to ensure the fertilization process. However, many animals are capable of asexual reproduction [3,4,5,6]. In this case, special germ cells and gonads are not required. Instead, reproduction relies on the separation of a body fragment from the maternal organism on the background of somatic cell proliferation. Agametic reproduction allows rapid expansion within distinct ecological niches, as well as effective multiplication even in the absence of a sexual partner. Some forms of asexual propagation are associated with the formation of resting stages, facilitating survival in adverse conditions, and may significantly contribute to the species’ distribution. As a result, asexual propagation plays an extremely important role in the life cycles of many animals [2,3,4,5,6]. In some cases, agametic reproduction leads to offspring that form gonads and germ cells. In syllids (the errant polychaete annelids), a peculiar version of fission (stolonization) gives rise to the individuals called stolons, which are filled with germ cells [3,6,7]. Often, such alternation of sexual and asexual generations leads to a change in morphological forms during the life cycle. For example, during medusoid budding or strobilation, the cnidarian asexual polyps ultimately produce free-swimming jellyfish or attached medusoids, which form gonads and are responsible for embryonic development. However, most often, a switch of reproductive mode does not involve a significant change in anatomy. Moreover, the transition from asexual to sexual reproduction, and vice versa, can occur without the alternation of generations [2,5]. The choice between different forms of reproduction is determined by various factors. The most important of these are nutrition, population density, temperature, and length of daylight. For aquatic inhabitants, the level of oxygen and organic contaminants is also of considerable importance. Many freshwater species that actively reproduce asexually in the summer, proceed to sexual reproduction in the autumn. The embryos of such animals can be well protected by cocoon-like shells and undergo a long diapause in development [5,8,9,10,11,12,13,14,15].

Interestingly, the processes of asexual reproduction and regeneration, on the one hand, and the formation of gonads, on the other hand, often compete with each other. That is, the animals stop breeding asexually after the onset of sexual maturation. Examples of gonad formation and/or active processes of gametogenesis occurring within a background of asexual reproduction are quite rare [3,4,5,6]. On the other hand, conserved germline markers [16,17,18], such as the homologs of *Vasa*, *Pl10*, *Piwi*, *Nanos,* and others, are involved in both embryonic (first of all, the germ cell line) [17,18,19,20,21,22,23,24,25] and postembryonic development, including regeneration and asexual reproduction [18,22,23,24,25,26,27,28,29,30,31,32,33,34,35].

Genes of germline multipotency program (GMP) are proposed to have a role in maintaining an undifferentiated state of cells and are required for the function of somatic pluri-/multipotent cells and germ cells in diverse animals [2,16]. These genes are mostly involved in the transcriptional repression of somatic programs, in chromatin reorganization, and in post-transcriptional regulation of several genes during development. It is believed that homologs of these genes were already present in the last common ancestor of metazoans [18] and that the urbilaterian germ cell multipotent progenitor was controlled by the GMP [16]. *Vasa*, *Pl10*, and *Piwi* are among the most conserved GMP genes. They belong to different gene families; however, mutations within these genes or gene knockout cause male sterility or female germ cell deficiency [2,16,17,18,20,35]. The expression of some of GMP genes has been investigated in a few annelids. *Vasa*, *Nanos*, and *Piwi* homologs are expressed in presumptive germ-cell precursors, and in developing mature gonad tissue [20,21,22,23,24,25,26,30]. In addition, *Vasa*, *Nanos*, *Pl10*, and *Piwi* have also been shown to be expressed in somatic tissues such as the posterior growth zone and regeneration blastema whose cells actively proliferate and exhibit a multipotent state (reviewed in [6,28,29]). Thus, GMP genes are useful for identifying germline cells and multipotent cells during gemetogenesis and and whole-body regeneration (including regrowth of a complete head or tail) in annelids.

Among annelid worms, naidids are distinguished by their ability to reproduce asexually [36,37,38,39]. They are small, often transparent animals inhabiting many freshwater bodies. Sexual animals are hermaphroditic. They develop gonads, a pair of testes and a pair of ovaries, in two consecutive segments, along with somatic structures of the oligochaete reproductive system [40,41]. In nature, sexually mature individuals are quite rare, usually found closer to autumn [15]. During the warm period, they actively proliferate by transverse division. Most naidids are not known to reproduce sexually under laboratory conditions [37,38,42,43].

In this study, we have shown that asexually reproducing individuals of naidid *Pristina longiseta* were able to establish germ cells and become fully sexually mature. We also have discovered that asexual propagation and germ cell/gonadal development/maturation are not mutually exclusive. We have identified five GMP genes, homologs of *Vasa* (*Plo-vasa*), *Piwi* (*Plo-piwi1*, *Plo-piwiA*, and *Plo-piwi2*), and *Pl10* (*Plo-pl10*), and characterized their expression using whole-mount in situ hybridization. During asexual reproduction and sexual maturation, *Vasa*, *Piwi*, and *Pl10* homologs are differentially expressed in both somatic and germline tissue including the posterior growth zone, fission zone, nervous system, germline cells, and gametes.

## 2. Materials and Methods

### 2.1. Animal Material and Fixation

The laboratory culture of *Pristina longiseta* was maintained using specimens originally found in a pond in the park of the Biological Institute of Saint-Petersburg State University (Russia) in 1999. Animals were cultured in Petri dishes with artificial spring water and Chlorophyta algae at 18 °C. Mashed spinach or dried spirulina powder was used as feed. As described previously [38], artificial illumination (16 h day, 8 h night) was used to optimize the intensity of asexual reproduction in cultures. We found that animals became occasionally sexualized under standard conditions for our laboratory culture. Thus, both fissioning and sexualized worms were collected from actively growing cultures.

Attempting to promote sexualization in *P. longiseta*, we have carried out a series of experiments with environmental shift. First, we have changed the photoperiod parameters by reducing daylight hours to 10 h (which corresponds to the length of the day in autumn or spring). In a series of parallel experiments, we varied the temperature by increasing it to 25 °C or decreasing it to 14 °C. Animals were observed every two days for 1 month. Worms continued to reproduce asexually, but showed no signs of mature gonads or gametes. The increased temperature led to the acceleration of asexual reproduction. Shorter daylight hours, as well as lower temperatures, slightly reduced the rate of population growth through asexual reproduction. Next, to assay the effect of starvation/refeeding, worms from the main laboratory culture were placed in separate Petri dishes with clean water. Three weeks later, these animals were fed. Starving animals stopped fissioning. After refeeding, they again began to reproduce asexually. Thus, the different conditions of these experiments affected the rate of asexual reproduction; the cues for initiation of sexual maturation are still unknown for *P. longiseta*.

To obtain materials for in situ hybridization and DIC analysis, worms were relaxed for 10 min in relaxant solution (10 mM MgCl_2_/5 mM NaCl/1 mM KCl/8% ethanol; see [44]) prior to fixation. For the DIC analysis of unlabeled intact objects, specimens were fixed overnight in 4% formaldehyde in 0.75× PBS/0.1% Tween-20 at 4 °C., and embedded in glycerol/PBS solution (9:1). For in situ hybridization experiments, the specimens were fixed in 4% PFA in PBS with 0.1% Tween 20 at +4 °C overnight and stored in MeOH at −20 °C.

### 2.2. Semi-Thin Sections Preparation

To prepare serial semi-thin sections, anesthetized fissioning worms were fixed in 2.5% glutaraldehyde in 0.1 M cacodylate buffer for 2.5 h, washed in the same buffer, and postfixed in 1% OsO4 in 0.1 M cacodylate buffer. The fixed material was dehydrated in ethanol and embedded in Epon (Fluka). Semi-thin sections were prepared on a Leica EM UC7 ultramicrotome, poststained with methylene blue, and embedded in Histokitt medium (Roth Chemie, Karlsruhe, Germany).

### 2.3. Gene Cloning and Phylogenetic Analysis

To identify *P. longiseta* homologs of *Vasa*, *Piwi*, and *Pl10*, PCR reactions with degenerate primers and asexually reproducing worms’ cDNA were performed. Extended fragments for *Plo-vasa*, *Plo-piwi1*, *Plo-piwiA*, *Plo-piwi2*, and *Plo-pl10* were amplified by 5′-RACE and/or 3′-RACE PCR with gene-specific primers and cDNA prepared with the SMARTer RACE cDNA Amplification Kit (Clontech, Mountainview, CA, USA). All primers are given in the Supplementary Materials (Table S1). These amplified gene fragments were cloned into pCRII vectors (TOPO-TA cloning, Invitrogen, Waltham, MA USA) that were used in the transformation of chemically competent *E. coli* (One Shot^®^ TOP10). Plasmids with correct inserts were sequenced and used for RNA probe synthesis. The identity of cloned sequences was confirmed through phylogenetic analysis. Amino acid translations of the five *P. longiseta* genes were aligned to homologs and outgroups from other species (Supplementary Materials, Tables S2 and S3) using MUSCLE 3.8.31 [45] at the Phylogeny.fr web server (accessed on 26 June 2023) [46]. Bayesian phylogenetic analysis was conducted using the Markov-chain Monte Carlo method implemented in MrBayes 3.2.6 (http://www.phylogeny.fr/ accessed on 26 June 2023) [46,47,48] as previously described [30]. The phylogenetic trees were handled using the FigTree program, v1.4.4 (http://tree.bio.ed.ac.uk/software/, accessed on 26 June 2023) (Supplementary Materials, Figures S1 and S2). The obtained sequences of *Plo-vasa*, *Plo-pl10*, *Plo-piwiA*, *Plo-piwi2*, and *Plo-piwi1* were deposited in GenBank with the accession numbers JX264563-JX264567, and OR203685.

### 2.4. Whole-Mount In Situ Hybridization

Whole-mount in situ hybridization (WMISH) was carried out as previously described [49]. Templates for the digoxigenin-labeled RNA probes (antisense and sense) were about 2500 bp (*Plo-vasa*), 1800 bp (*Plo-pl10*), 980 bp (*Plo-piwi1*), 2800 bp (*Plo-piwiA*), and 830 bp (*Plo-piwi2*). After hybridization with antisense digoxigenin-labeled RNA probes, incubation with anti-digoxigenin AP antibodies (1:2500, Roche, Mannheim, Germany), staining with NBT/BCIP, and washing, specimens were mounted in 90% glycerol. In situ hybridization with the sense DIG-labeled riboprobes was used as a negative control.

### 2.5. Data Visualization

Imaging of the mounted in-glycerol or in-Histokitt-medium specimens was conducted using DIC optics on an Axio Imager D1 microscope (Carl Zeiss, Oberkochen, Germany) equipped with digital camera AxioCam ICc5 (Carl Zeiss, Oberkochen, Germany). Pictures were acquired and edited with programs AxioVision 4.8 and Adobe Photoshop CS5.1.

## 3. Results

### 3.1. Asexual Reproduction in Pristina longiseta 

**Figure 1:**
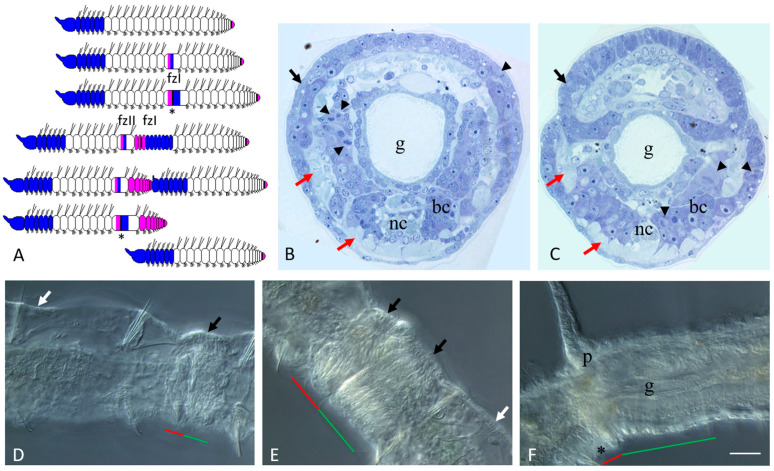
Schema of rapid paratomy and different stages of asexual reproduction in *Pristina longiseta*. (**A**) Schema of rapid paratomy in *P. longiseta*. The first fission zone (fzI) usually appears within a segment within from 14 to 18, and gives rise to a new cephalic (head) region, and a new tail end with pygidium, growth zone (*), and some trunk segments. During progressive formation of fission zone I (fzI), an additional second fission zone (fzII) becomes evident in more anterior segment. After the new anterior and posterior ends of two-zooid development, the paratomy process is continued by the physical separation of the two daughter individuals. The blue color marks the head region and cephalogenic part of the fission zone; the pink color marks the somatogenic part of the fission zone and its derivatives. (**B**,**C**) Cross semi-thin sections through the cephalogenic part of the fission zone (**B**) and at the level of the boundary between the anterior and posterior zooids (**C**). Epidermal cells are modified and have morphology similar to the blastemal cells. Staining with methylene blue. (**D**–**F**) Fission zone at different stages of asexual reproduction in Pristina longiseta, DIC. Early middle stage (**D**) and middle stage (**E**) of fission-zone development. Both parts of fission zone, cephalogenic part and somatogenic, become evident. (**F**) Late stage of fission zone development. p—prostomium, bc—blastemal cell mass, g—gut, nc—ventral nerve cord. Arrowheads indicate some of dividing cells. Black and white arrows show modified and intact integumentary epithelium, respectively. Red arrows point to body wall muscles. The red line marks the new developing tail region, the green line marks the new developing head region within the fission zone, and the asterisk marks the posterior growth zone of anterior zooid. Scale bar in (**B**,**C**) 25 µm. Scale bar in (**D**–**F**) 45 µm.

Ready to undergo paratomic fission, *Pristina longiseta* worms typically comprise 21–29 segments. A cephalic region consisting of the prostomium, peristomium, and specialized head (cephalic) segments is present at the anterior end of the worm. The posterior growth zone, that produces new segments during normal growth, is located just anterior to the pygidium at the posterior end. The number of head segments is constant and species-specific. In *P. longiseta*, it comprises six segments. The head segments are distinguished from all other segments by the absence of chloragogen cells and nephridia. Fission zones are typically formed between segments 14 and 18. Thus, there is no fixed segment for developing the paratomic zone (see also [38,49] for more details). Agametic reproduction in *P. longiseta* occurs by paratomy. It means that the new anterior and posterior ends of the two zooids develop prior to the physical separation of the fusioning individual (Figure 1).

The paratomic fission zone forms in the one-third of a segment behind the anterior border of a trunk segment. Later, it is subdivided by a circular epidermal thickening into two parts, the cephalogenic and somatogenic parts (Figure 1A,D,E). The cephalogenic part gives rise to the new cephalic region of the posterior zooid, and the somatogenic part gives rise to the new posterior end (with pygidium and growth zone) of the anterior zooid. The physical separation of the two daughter individuals occurs only after the complete formation of all these structures (Figure 1F). Under optimal conditions, a worm can develop multiple fission zones, and every additional fission zone is usually initiated in progressively more anterior segments. Thus, *P. longiseta* reproduces by so-called rapid paratomy [5].

**Figure 2:**
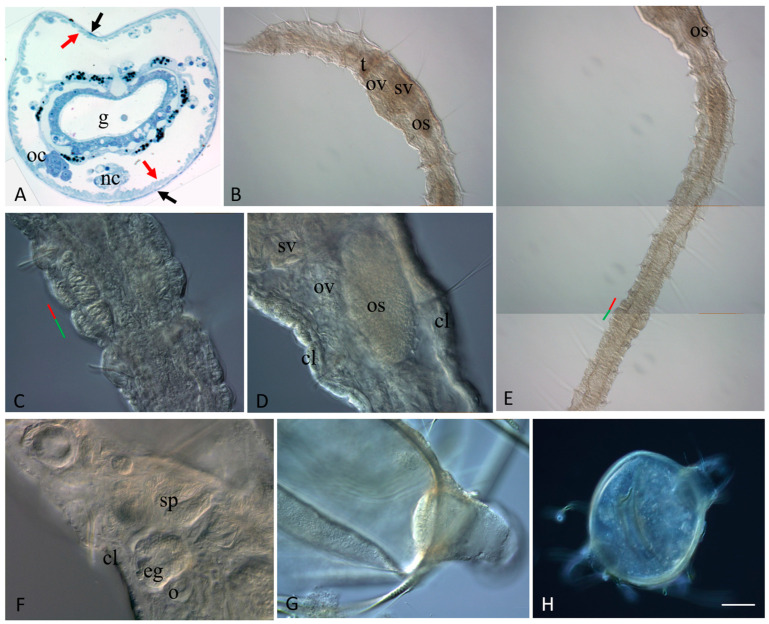
Gonad anlage and sexualized *P. longiseta* worms. In (**B**–**F**), the anterior is up. (**A**) Cross semi-thin section through the segment VII of the posterior zooid of an asexually reproducing worm, before physical separation of new individuals from each other. Black and red arrows show intact integumentary epithelium and body wall muscles, respectively. Staining with methylene blue. (**B**,**E**) A sexualized individual of *P. longiseta*, anterior (**B**) and trunk (**E**) regions, DIC. (**C**) Developing fission zone of the sexual mature *P. longiseta* worm showed in (**B**,**E**), early stage. (**D**) Another specimen of sexualized worm with a large egg in ovisac, DIC. (**D**) Germ cells in a mature *Pristina longiseta* specimen, DIC. (**G**,**H**) The cocoons of *P. longiseta*, DIC. cl—clitellum, eg—egg floating in coelomic cavity, o—growing oocyte, oc—cluster of oocyte, os—ovisac, ov—ovaries, t—testis, sp—sperm, sv—seminal vesicle. The red line marks the new developing tail region, and the green line marks the new developing head region within the fission zone. Scale bar: 25 µm (**A**), 45 mkm (**C**,**D**,**F**,**G**), 70 µm (**B**,**E**), and 120 µm (**H**).

At the early and middle stages of development, the paratomy zone differs significantly from other parts of the body in cell composition (Figure 1B–E). It lacks such differentiated cell types such as chloragogen cells and cells of nephridia, while muscle fibers become modified and gradually break during fission (Figure 1B,C and Figure 2A; see also [38,41] for more details). Only the intestinal epithelium remains seemingly unchanged. At the same time, a significant number of small, undifferentiated cells with a high nuclear–cytoplasmic ratio and intensively basophilic cytoplasm were observed within the fission zone on the semi-thin sections (Figure 1B,C). Some of these cells proliferated (Figure 1B,C). The integumentary epithelium of the paratomy zone undergoes significant modifications as well. After staining with methylene blue, the cytoplasm of epithelial cells in the paratomy zone was more basophilic than in other areas (Figure 1B,C and Figure 2A). These cells were columnar and drop-shaped instead of the normal flattened morphology. Many cells displayed mitotic activity. The boundaries between the modified epithelium and the mass of deep undifferentiated cells were not obvious, and cells of these two domains were not clearly distinguished from each other.

Later, when an external epithelial furrow grows, and a constriction between the anterior (somatogenic) and posterior (cephalogenic) cell masses of the fission zone become evident (Figure 1C), structures of new cephalic region begin to form (Figure 1G,E) including the presumptive prostomium, as a growing epidermal fold, and the primordia of the cerebral ganglion, as a derivate of deep undifferentiated cells. The cephalogenic mass of deep cells, having increased greatly in size, segments to form bilateral pairs of chaetal sacs. Simultaneously, the posterior growth zone of the anterior zooid begins to produce new trunk segments (Figure 1F). 

During the final growth of the paratomic zone, the old segments behind the new head display a segment identity shift. The gut in the segment immediately behind the fission zone thickens and forms a new stomach by morphallaxis [28,44,49,50]. Another important manifestation of this shift is the establishment of female gonad-like structures in previously non-gonadal segments (Figure 2A).

By analyses of the semi-thin sections through the VII segment of the posterior zooid (an old segment that became the first trunk segment), the ovary-like structures were found on both sides of the ventral nerve cord close to the anterior septum. Each ovary was composed of germ and somatic cells in close association (Figure 2A). The germ cells were interpreted as oogonia. At the same time, in the most posterior new-formed cephalized segment of the posterior zooid (segment VI), some male-specific structures became evident (see below). 

We also found that many components of male and female reproductive systems are usually reduced or absent in the worms that actively propagated asexually.

### 3.2. Sexually Matured Form of Pristina longiseta 

Naidid species in the wild reproduce both asexually and via sexualized individuals [5,15] but they are mostly not known to reproduce sexually under laboratory conditions [37,38,42,43]. For example, *P. longiseta* worms were successfully propagated asexually only over a period of time of observation in our laboratory. Attempting to promote sexualization in *P. longiseta*, we have carried out a series of experiments with environmental shift. In particular, we have changed the photoperiod parameters as well as temperature and food availability. The different conditions of these experiments affected the rate of asexual reproduction but we were not able to induce sexualization and obtain fully mature individuals. Nevertheless, we found over forty sexualized individuals in several Petri dishes of our laboratory cultures. These mature worms were used for both morphological and molecular research. These specimens became sexualized under standard conditions for our laboratory culture. Thus, the cues for initiation of sexual maturation are still unknown for *P. longiseta*. All these sexualized worms demonstrated various stages of maturation. Most of them developed sexual anatomy (Figure 2B,D–F). Sexualized worms were hermaphroditic and developed variable-in-size gonads in two consecutive segments, a pair of testes in segment VI, and a pair of ovaries in segment VII. The fully matured animals developed a complete set of sexual apparatus including spermatheca, atrium, seminal vesicles, and ovisac. They also had a clitellum, the collar of thickened epithelium around the sexual segments. Germ cells (sperm and oocytes) were found inside the corresponding parts of the reproductive system in these specimens (Figure 2F). Altogether, the data suggest that the laboratory strain of *P. longiseta* maintains the ability to become fully sexually mature and to establish germline products even after a long period of agametic reproduction. During the intensive search, we found several egg- and ellipsoid-shaped cocoons (Figure 2G,H), but all of them were empty, without an egg or embryo. Thus, the question if these animals are really able to develop embryonically is still open.

Interestingly, many sexualized individuals undergo paratomic fission at the same time (Figure 2C,E). The animals showed various stages of fission-zone formation. Most of them were at the early or middle stages, but several were at the late stage and ready for the physical separation of the two daughter individuals. By this way, the processes of asexual reproduction and sexual maturation do not compete with each other. On the other hand, although the animals showed various stage of fission-zone formation, no one sexualized worm had multiple fission zones developing at once. 

### 3.3. Vasa, Piwi, and Pl10 Homolog Expression in Growing Adults and Asexually Reproducing Pristina longiseta 

One of the main goals of this study was to investigate how germline/multipotency markers are expressed during paratomic fission and sexual maturation in the annelid *P. longiseta*. In this work, we identified in *P. longiseta* three homologs of *Piwi* (*Plo-piwi1*, *Plo-piwiA*, and *Plo-piwi2*), one homolog of *Vasa* (*Plo-vasa*), and one homolog of *PL10* (*Plo-pl10*), and examined their developmental patterns by WMISH.

**Figure 3:**
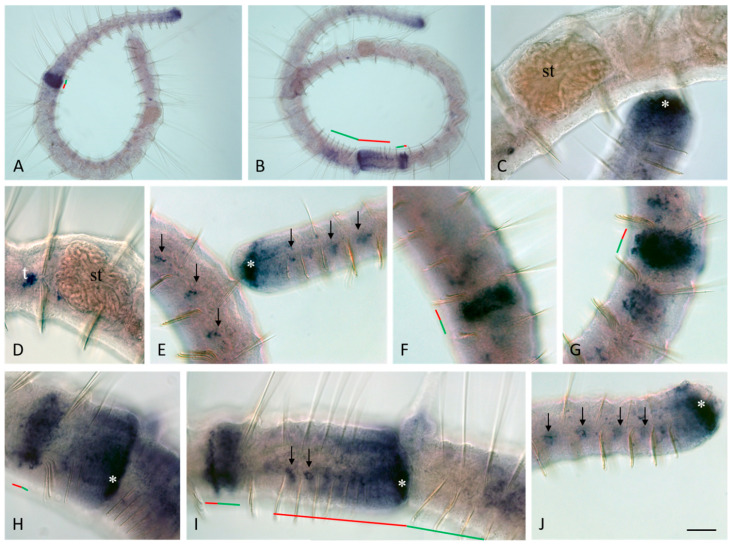
*Plo-vasa* expression during asexual reproduction in *P. longiseta*. Except for (**A**,**F**,**G**), all animals are oriented anterior to the left, while in (**A**,**F**,**G**), anterior is up. Lateral view for all panels. *Plo-vasa* is strongly expressed at the posterior end of the worms, in somatic tissue of the young segments and posterior growth zone, but not in the pygidium, in which no expression is detected (**A**,**E**,**C**,**J**). *Plo-vasa* appears to be de novo expressed in developing fission zone and is shown at various stages of asexual reproduction, from the earliest steps until physical separation of new individuals each from other (**A**,**B**,**F**–**I**). Arrows indicate additional domains of internal patches of *Plo-vasa*-positive cells in middle and posterior trunk segments (**E**,**I**,**G**). *Vasa* transcripts are found in anlage of gonads, testes and ovaries (**C**,**D**). st—stomach, t—testis. The asterisk marks the posterior growth zone, the red line marks the new developing tail region, and the green line marks the new developing head region within the fission zone. Scale bar, 45 µm for all panels except (**A**,**B**). Scale bar in (**A**,**B**), 70 µm.

**Figure 4:**
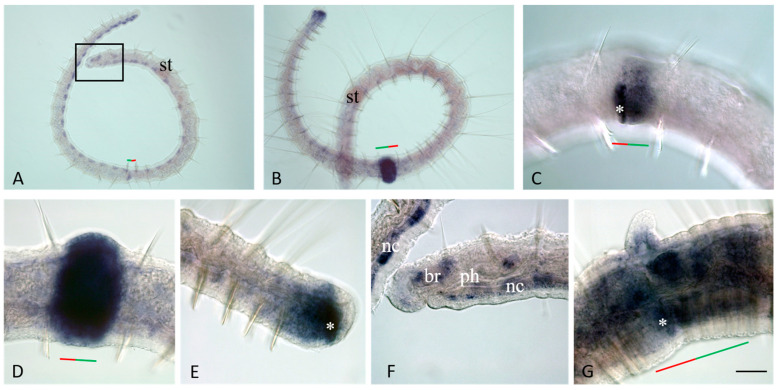
*Plo-pl10* expression during asexual reproduction in *P. longiseta*. All animals are oriented anterior to the left, lateral view. (**A**) Early-fission stage. (**B**–**D**) Mid-fission stage. (**G**) Late-fission stage. *Plo-pl10* is strongly expressed in somatic tissue of the young segments and posterior growth zone; no expression is detected in the pygidium (**A**,**B**,**E**) and appears to be de novo expressed in developing fission zone (**A**–**D**,**G**). *Plo-pl10* shows its activity in the nervous system, including ventral nerve cord and brain, as well as in circumpharyngeal domain. (**F**) Expression in the head region of an intact worm. br—brain, nc—ventral nerve cord, ph—pharynx, st—stomach. The asterisk marks the posterior growth zone, the red line marks the new developing tail region, and the green line marks the new developing head region within the fission zone. Scale bar, 45 µm for all panels except (**A**,**B**). Scale bar in (**A**,**B**), 70 µm.

**Figure 5:**
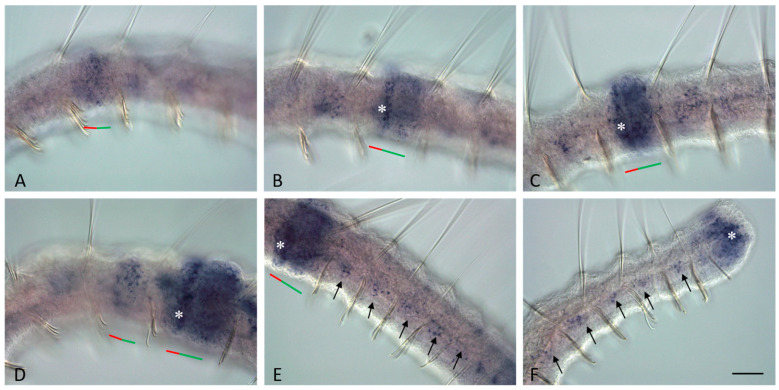
*Plo-piwi1* expression during asexual reproduction in *P. longiseta*. All animals are oriented anterior to the left. Lateral view for all panels. *Plo-piwi1* appears to be expressed de novo in the developing fission zone and transcripts of this gene are shown at various stages of asexual reproduction, from the earliest steps to the latest stage (**A**–**E**). Expression is observed in an additional fission zone (**D**). *Plo-piwi1* is also strongly expressed at the posterior end of the worms, in somatic tissue of the young segments and posterior growth zone, but not in the pygidium, in which no expression is detected (**F**). Arrows indicate additional domains of internal and superficial patches of *Plo-piwi1*-positive cells in middle and posterior trunk segments (**E**,**F**). The asterisk marks the posterior growth zone, the red line marks the new developing tail region, and the green line marks the new developing head region within the fission zone. Scale bar, 45 µm for all panels.

**Figure 6:**
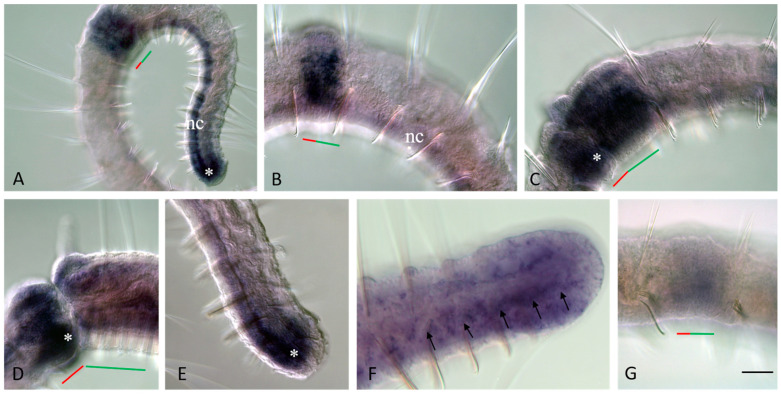
*Plo-piwiA* and *Plo-piwi2* expression during asexual reproduction in *P. longiseta*. Except for (**E**), all animals are oriented anterior to the left, while in (**E**), anterior is up. Lateral view for all panels. *Plo-piwiA* appears to be expressed de novo in the developing fission zone and transcripts of this gene are shown at various stages of asexual reproduction, from the earliest steps to the latest stage (**A**–**D**). *Plo-piwiA* is also strongly expressed at the posterior end of the worms, including somatic tissue of the young segments, posterior growth zone and in the posterior part of the ventral nerve cord (**A**); no expression is detected in the pygidium (**A**,**E,F**). Its transcripts are shown in the posterior part of the ventral nerve cord (**A**). Arrows indicate additional domains of internal *Plo-piwiA*-positive cells in the posterior trunk segments, including cells underlying the gut ((**F**), deep focal plane). (**G**) *Plo-piwi2* is de novo expressed at a very low level during the early mid stage of fission zone formation. The asterisk marks the posterior growth zone, the red line marks the new developing tail region, and the green line marks the new developing head region within the fission zone. Scale bar, 45 µm for all panels except (**A**) and (**F**). Scale bar in (**A**), 70 µm. Scale bar in (**F**), 35 µm.

*Plo-piwi2* is the only gene whose mRNA is not detected by in situ hybridization in growing adult *P. longiseta* worms. All other genes show strong expression in the superficial and deep cells of young segments and the posterior growth zone in both growing adult worms and asexually reproducing animals. The expression domains of *Plo-vasa*, *Plo-piwi1*, *Plo-piwiA*, and *Plo-pl10* are especially wide on the lateral and ventral sides; however, *Plo-piwiA* signal is more diffuse. None of these gene transcripts were detected in the pygidium (Figure 3A,B,E,J, Figure 4A,B,E, Figure 5F and Figure 6A,E). *Plo-piwiA* and *Plo-pl10* transcripts were found in ventral-nerve-cord cells (Figure 4A,B,F and Figure 6A,E). In addition, *Plo-piwiA*-positive cells were also seen in the hindgut region (Figure 6F). 

In segments of the animal’s body without a fission zone, the expression of *Plo-vasa* and *Plo-piwi1* shows a character that is more complex. In the posterior third of the body of *P. longiseta*, the domains of *Plo-vasa* and *Plo-piwi1* expression look like small bilaterally (mostly ventrolaterally) located patches of cells of the integumentary epithelium or internal cells (Figure 3E–G,I,J and Figure 5C,E). The anterior border of such an expression pattern corresponds typically to the position of a fission zone or is located slightly anteriorly.

**Figure 7:**
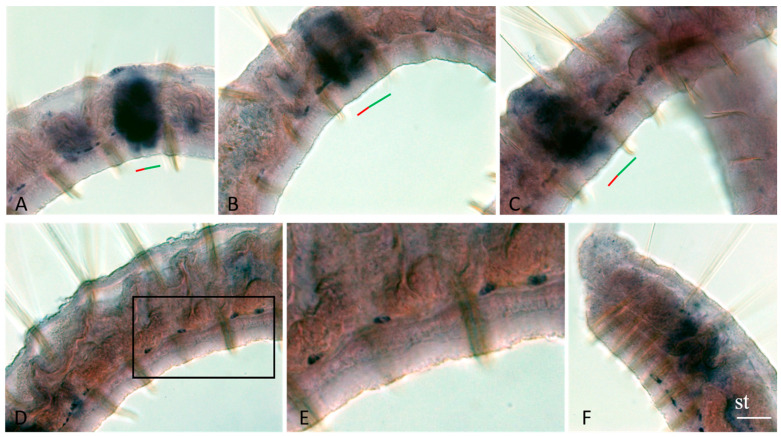
*Plo-vasa* expression in the cells along the ventral nerve cord in asexually reproducing worms. Except for (**F**), all animals are oriented anterior to the left, while in (**F**), anterior is up. Lateral view for all panels, deep focal plane. *Plo-vasa*-positive ventral cells, various in number, are shown on the ventral nerve cord within the trunk segments. (**A**–**C**) Middle trunk segments. (**D**) More posterior trunk segments. (**E**) Enlarged view of the boxed region shown in (**D**). (**F**) the most anterior trunk segments. st—developing stomach in new individual (posterior zooids). The red line marks the new developing tail region, and the green line marks the new developing head region within the fission zone. Scale bar, 45 µm for all panels except (**E**). Scale bar in (**E**), 15 µm.

**Figure 8:**
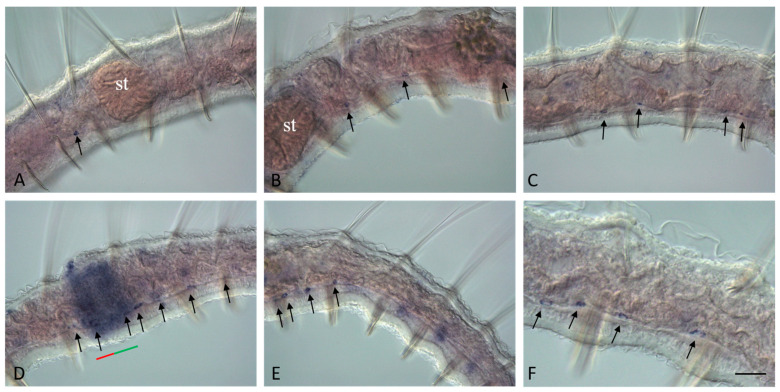
*Plo-piwi1* expression in the cells along the ventral nerve cord in asexually reproducing worms. All animals are oriented anterior to the left. Lateral view for all panels, deep focal plane. *Plo-piwi* positive ventral cells (arrows), various in number, are found on the ventral nerve cord within the trunk segments. (**A**,**B**) Anterior trunk segments. (**C**,**D**,**F**) Middle trunk segments. (**E**) More posterior trunk segments. (**F**) A magnified view of the mid-body region. St—stomach. The red line marks the new developing tail region, and the green line marks the new developing head region within the fission zone. Scale bar, 45 µm for all panels except (**F**). Scale bar in (**F**), 18 µm.

Along the ventral nerve cord, dorsally to it, there are single *Plo-vasa*- and *Plo-piwi1*-positive cells. These cells are spindle-shaped and have a high nuclear–cytoplasmic ratio. The most anterior localization of such cells is at the level of the first postlarval (trunk, not head) segment. The number of such cells in the segments, as well as in different samples, varies greatly and may correlate with the feeding conditions of the animals (Figure 7 and Figure 8).

*Plo-vasa*, *Plo-pl10*, *Plo-piwi1*, *Plo-piwiA*, and *Plo-piwi2* are expressed de novo in the area of paratomy (Figure 3, Figure 4, Figure 5 and Figure 6). *Plo-vasa* appears to be expressed earlier than other genes in this area, being present, already, at the very early stages of development of the fission zone (Figure 3F). *Plo-pl10* and *Plo-piwi1* transcripts appear within the paratomy zone a little later and *Plo-piwiA* occurs even later and less intensively. A very low level of diffuse *Plo-piwi2* expression can only be detected at the early mid-fission stage (Figure 6G). The first signs of expression of *Plo-vasa*, *Plo-pl10*, *Plo-piwi1*, and *Plo-piwiA* appear in the cells of the modified epidermis; then, as blastemal masses develop, large expression domains are formed corresponding to the internal masses of undifferentiated cells. The expression level remains very high throughout the middle stage and the beginning of the late paratomy in both the developing new caudal end and the developing head region. High levels of *Plo-vasa*, *Plo-pl10*, *Plo-piwi1*, and *Plo-piwiA* transcripts are seen in the posterior growth zone of the anterior zooid (Figure 3G–I, Figure 4G, Figure 5D and Figure 6C,D), during the late stage of asexual reproduction, a distinct domain of *Plo-pl10* expression appears in the intestine at the level of the future seventh segment of the posterior zooid. The expression of all these genes gradually disappears in the cells of the integumentary epithelium, and then in the internal cells of the cephalogenic part of the fission zone. Nevertheless, low levels of *Plo-vasa* and *Plo-pl10* transcripts are found in the head region of the posterior zooid immediately after physical separation from the anterior (parent) individual (Figure 7F). At the end of morphallactic remodeling of the gut into the stomach (segment VII), *Plo-vasa* expression is no longer detected in the head region, but *Plo-pl10* continues to be expressed in the anterior nerve ganglia, as well as in a group of cells dorsally and laterally adjacent to the cerebral ganglia and buccal pharynx (Figure 4F,J).

### 3.4. Vasa, Piwi, and Pl10 Homolog Expression in Sexually Matured Pristina longiseta 

In actively asexually reproducing *P. longiseta*, small ventrolateral clusters of *Plo-vasa-*, *Plo-pl10-*, and *Plo-piwi1*-positive cells are found close to the anterior septum of segment VI, and sometimes segment VII. According to the available data on the structure of the reproductive system and gametogenesis in oligochaetes, naidids in particular [40,41], the anlages of the testes and ovaries appear in segments VI and VII, respectively, on the border of the larval (head region) and postlarval body. During *P. longiseta* sexual maturation, the testes increase in size; the clitellum, seminal and ovarian vesicles, and other structures appear. The germ cells of oligochaetes leave the gonads very early. Clusters of 8–16 spermatogonia, which are products of incomplete cytokinesis, separate from the testes. They enter the body cavity and then into the seminal vesicles. Spermatogonia in each cluster actively proliferate, and as a result, large masses of cells are formed. In the ovaries, the development of female germ cells occurs up to the stage of early oocytes, which also form groups of 16–32 cells. Further development of female germ cells occurs in the coelom of the ovarian segment, and later in the ovisac. In each of the groups of oocytes, only one cell becomes an egg. It accumulates yolk and undergoes meiosis. Thus, the gonads themselves do not exist for long and quickly disappear, especially in naidids. Male gonads are always formed earlier than female gonads (protandric hermaphroditism) [40,41].

**Figure 9:**
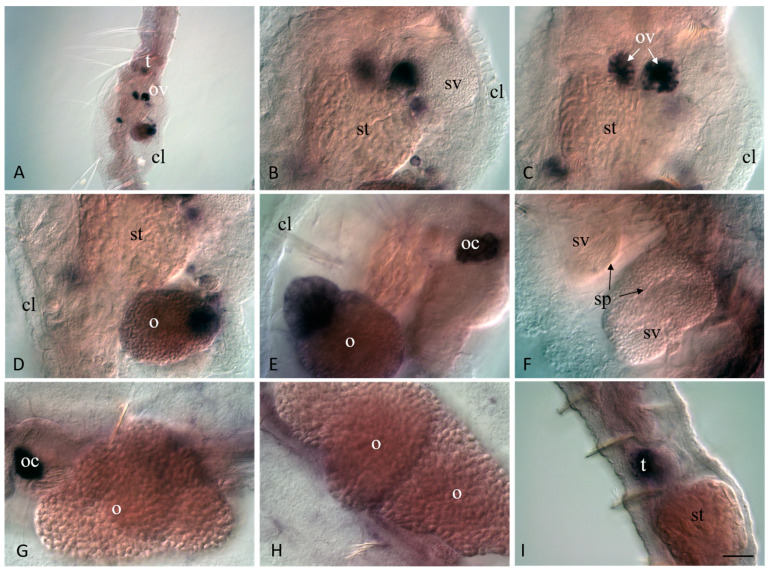
*Plo-vasa* expression in sexually mature *P. longiseta*. Except for (**G**), all animals are oriented anterior to the up direction, while in (**G**), anterior is on the left. Lateral view for all panels, deep focal planes. (**B**–**D**) Enlarged view of the gonadal segments of the animal shown in (**A**). (**E**–**H**) Another sample of sexually mature worms at the late stages of gametogenesis. (**D**) An example of the gene expression at early-to-mid stage of testis development. (**I**) An example of *Plo-vasa* expression at early stage of testis development. cl—clitellum, o—growing oocyte, oc—cluster of oocyte, ov—ovaries, t—testis, sp—sperm, st—stomach, sv—seminal vesicle. Scale bar, 45 µm for all panels except (**A**). Scale bar in (**A**), 70 µm.

**Figure 10:**
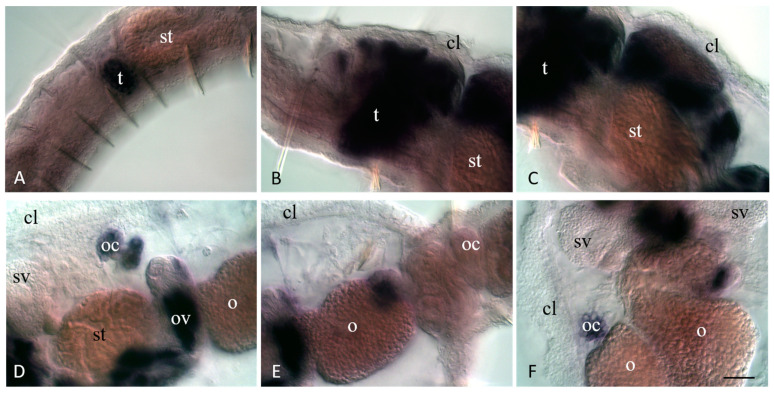
*Plo-pl10* expression in gonadal segments of the sexually mature *P. longiseta* worms. Except for (**F**), all animals are oriented anterior to the left, lateral view; in (**F**), anterior is up, ventral view. Deep focal planes for all panels. Animals are at a different stage of sexual maturation: early stage (**A**), mid stage (**B**,**C**), and late stage (**C**–**F**). cl—clitellum, o—growing oocyte, oc—cluster of oocyte, ov—ovaries, t—testis, st—stomach, sv—seminal vesicle. Scale bar, 45 µm for all panels.

**Figure 11:**
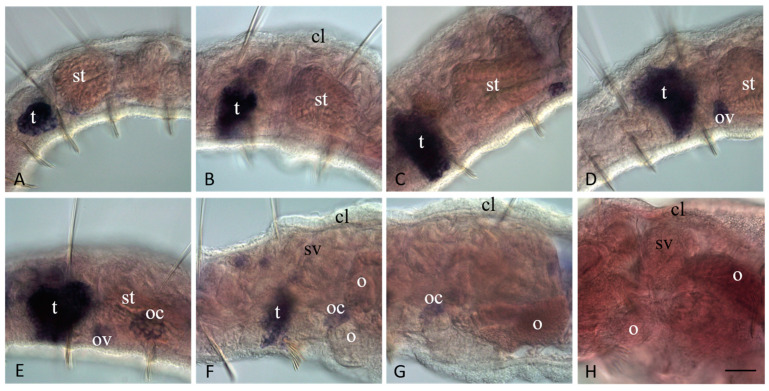
In contrast to *Plo-piwiA*, *Plo-piwi1* is expressed in the gonadal segments of the sexually mature *P. longiseta* worms. All animals are oriented anterior to the left. Lateral view for all panels, deep focal planes. Animals are at a different stage of sexual maturation: early stage (**A**), mid stage (**B**–**E**), and late stage (**F**–**H**). mRNA of *Plo-piwi1* is detected in testes and clusters of male and female germ cells; *Plo-piwi1* expression disappears at the late gametogenesis (**F**–**G**). No expression of *Plo-piwiA* is detected at any stage of sexual maturation of *P. longiseta*, including late stage (**H**). cl—clitellum, o—growing oocyte, oc—cluster of oocyte, ov—ovaries, t—testis, st—stomach, sv—seminal vesicle. Scale bar, 45 µm for all panels.

The results of in situ hybridization show the differential character of the active expression of the *Plo-vasa*, *Plo-pl10*, and *Plo-piwi1* genes at the stages of gonadal development and functioning (Figure 9, Figure 10 and Figure 11). mRNAs of these genes are detected already at the earliest stages of male germ-cell formation. A very high level of transcripts is characteristic of the *Plo-pl10* gene, which is observed, probably, until the stage of spermiogenesis, i.e., the completion of spermatozoa formation in the seminal vesicles (Figure 10A–C). mRNA of other genes disappears in spermatocytes much earlier. Expression of *Plo-piwi1* fades in cell clusters that have left the testes, and *Plo-vasa* expression is limited by the time of formation of such clusters in the testes. No gene expression was detected in spermatozoa (Figure 9A,D,F, Figure 10F and Figure 11F).

*Plo-vasa*, *Plo-pl10*, and *Plo-piwi1* are expressed early in the development of female germ-cell clusters (Figure 9C, Figure 10D and Figure 11C–E). *Plo-vasa* and *Plo-pl10* transcripts were also found in oocytes floating in the coelomic cavity and in yolk-rich eggs (Figure 9D,E,G,H and Figure 10E,F). In contrast, *Plo-piwi1* expression does not last long in oocytes and is not detected in yolk-rich eggs (Figure 11C–G).

During *P. longiseta* sexual maturation, *Plo-piwiA* expression is not associated with either gonadal development or germ cell establishment and maintenance (Figure 11H).

## 4. Discussion

### 4.1. Reproductive Strategies in Pristina longiseta

Although virtually all multicellular animals are capable of embryonic development, many of them, from sponges to placental mammals, are also capable of asexual reproduction [2,3,4,5,6,11,14,18,27,29,35,39]. The very phenomenon of disintegration of the whole organism, in which an individual physically divides its body, and the acquisition by its parts of the status of individuals remains largely incomprehensible at the contemporary level of biology. The variety of forms of asexual reproduction and their distribution across taxa is evidence of independent repeated acquisition of this ability in different taxa [3,5,51]. Oligochaetes of the family Naididae are convenient models to study morphogenetic and evolutionary aspects of this problem. It is shown that in these worms, the paratomy type of transverse division occurred in several evolutionary lines on the basis of regenerative abilities [39,52]. In oligochaetes, paratomic fission is represented by two forms: slow paratomy and rapid paratomy. Slow paratomy is accompanied by the formation of chains from no more than two zooids (for example, *Nais communis*), while rapid paratomy leads to the formation of chains from several zooids (*P. longiseta*) [38,49]. In the case of *P. longiseta*, we observe one of the extreme forms of transition from sexual to predominantly asexual reproduction. In most other relevant examples, these forms of reproduction are in competitive relationships, i.e., individuals typically exhibit only one reproductive mode (agametic propagation or sexual reproduction) at a time [5,41,53]. Our work has illustrated this phenomenon in a new way. In *P. longiseta*, we found the initiation of gonad development in the segment of the body adjacent to the fission zone. The appearance of cell clusters, interpreted as prospective gonads, has also been described in a closely related species, *P. leidyi* [42,43]. It is noteworthy that the formation of clusters of gametogenic cells occurs in the old segment of the body undergoing morphallaxis. Such a restructuring of old tissues is much more complex and different from embryogenesis [1]. On the other hand, although tissue remodeling by morphallaxis occurs during both fission and sexual maturation, the mechanisms by which this is achieved should differ in the two contexts in naidid species. First, gonads are formed de novo by morphallaxis of previously non-gonadal segments. Second, development of reproductive system structures can be regulated under environmental and endogenous control, probably via endocrine regulation, and finally, the germ cells usually have a specific origin [27,28,54].

In our case, it was possible to find not only the initiation, but also a quite complete development of the sexual organ system. The appearance not only of mature gametes, but also clitellum and cocoon shells confirm this acquisition of sexual maturity. It is noteworthy that this spontaneous event occurred simultaneously in a large number of individuals that resembles the seasonal peak of sexual reproduction in other naidids [15]. Although a common pattern is that agametic propagation occurs in animals that are not yet sexually mature, and that such animals completely stop asexual reproduction before reproducing sexually, *P. longiseta* demonstrated the ability for both reproductive modes simultaneously. Thus, the processes of asexual reproduction and sexual maturation are not mutually exclusive in these animals. On the other hand, we did not find any embryos, and these data could be evidence of possible suppression of gamete formation (at final stages) or embryogenesis in the animals under laboratory conditions. In future studies, it will be worthwhile to determine the cues for the initiation of sexual maturation in *P. longiseta* to answer the question whether these animal are capable of embryonic development. 

Our data on *P. longiseta* corroborate older descriptions and showed that the ovaries in naidids are inconspicuous and germ cells detach from the ovaries and form groups of cells that float within the coelomic cavity and ovisac [40,41,55,56]. The fact that only the very beginning of oogenesis occurs in the ovaries suggest the organization of the female gonads in naidids differs essentially from that found in other oligochaete annelids [57]. 

### 4.2. Vasa, Piwi, and Pl10 Homolog Expression in the Context of Reproductive Strategies

Conserved germline markers, so-called germline multipotency program (GMP) genes [16,17,18,58,59], are strong molecular markers of germline across metazoans and can thus be useful tools for studies of germline establishment, germline stem-cell maintenance, and/or gametogenesis in a variety of developmental contexts. On the other hand, they are frequently expressed in multipotent cells, both somatic and germline, and might be required for regeneration and asexual reproduction [17,18,22,23,24,25,28,29,30,31,32,42,43,52,60]. Thus, expression of *Piwi*, *Vasa*, *Pl10*, and *Nanos* homologs has been observed in proliferative tissues in juveniles and adults of *Alitta virens* (*Piwi*, *Vasa*, *Pl10*, and *Nanos* homologs), *Platynereis dumerilii* (many GMP genes, including *Piwi*, *Vasa*, and *Nanos* homologs), *Capitella teleta* (*Piwi*, *Vasa*, and *Nanos* homologs) *Enchytraeus japonensis* (two *Vasa* homologs), and *P. leidyi* (*Piwi*, *Vasa*, and *Nanos* homologs) [22,23,24,25,30,31,43,58,60]. These results suggest that the expression of GMP genes in the posterior growth zone, fission zone, and regeneration blastema is an ancestral feature for annelids. Moreover, recent work has shown that *piwi*-positive cells can give rise to variable differentiation patterns in adult *P. leidyi* worms [61], raising even more intriguing questions.

**Figure 12:**
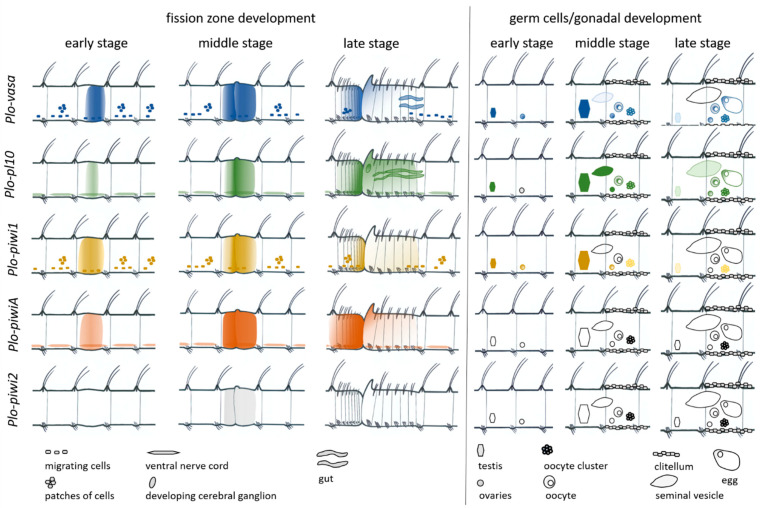
Summary of gene expression during *P. longiseta* asexual reproduction (fission zone formation) and sexual maturation (germ cells/gonadal development). Schematic representation of *Plo-vasa*, *Plo-pl10*, *Plo-piwi1*, *Plo-piwiA,* and *Plo-piwi2* expression patterns is shown in different colors for each gene. Lateral view, anterior to the left for all panels. During asexual reproduction, a region of new tissue referred to as a fission zone forms within a mid-body segment, developing a new tail and a new head. All five are expressed de novo in the area of paratomic fission zone. *Plo-vasa* and *Plo-piwi1* are also expressed in ventrolateral patches of cells (internal and epidermal), as well as in cells that are distributed along the ventral nerve cord and are likely migrating and associated with gonads. *Plo-vasa*, *Plo-pl10*, and *Plo-piwi1* genes are differentially expressed at the stages of gonadal development and gametogenesis, while transcripts of *Plo-piwiA* and *Plo-piwi2* are not detected in the gonadal segments (VI and VII) during the sexual maturation of animals. See text for more details.

Our results are consistent with data on the involvement of the *Vasa*, *Pl10*, and *Piwi* homologs in the formation of new proliferative tissues, such as the posterior growth zone and the fission zone [6,24,31,43,60,62]. Moreover, we have shown that *P. longiseta* has three homologs of *Piwi*, and that all three are expressed in the fission zone, although *Plo-piwi2* expression is very weak and transient. In contrast, in *P. leidyi*, only one of the two *Piwi* homologs is expressed in the fission zone [43]. In this work, we showed, for the first time, the involvement of the *Pl10* homolog in the formation (and thus, presumably, functioning) of the posterior growth zone and fission zone. In addition, this gene seems likely to be important for morphallactic remodeling of the gut during stomach formation in the posterior zooid, and can also have some function in the nervous system (Figure 12).

In *P. longiseta*, we found that two of the five GMP genes we investigated, *Plo-vasa* and *Plo-piwi1*, are expressed in isolated spindle-shaped cells distributed along the ventral nerve cord. The morphology of these cells suggests they are migrating cells. Similar *Piwi*-positive cells were also shown in *P. leidyi*; however, such *Vasa*-positive cells were undetectable by WMISH in this species. These cells appear to be associated specifically with the fission process and gonads [42,43].

*Plo-vasa*, *Plo-pl10*, and *Plo-piwi1* genes are differentially expressed at the stages of gonadal development and gametogenesis (Figure 12). As in *P. leidyi*, the expression of these genes suggests the development of prospective gonads, both testes and ovaries, in asexually reproducing *P. longiseta*. The expression of these genes is very characteristic for male germ-cell formation. The *Plo-pl10* gene is expressed in a prolonged period form the earliest steps of testis formation until the stage of spermiogenesis, i.e., completion of spermatozoa formation in seminal vesicles. *Plo-vasa* and *Plo-pl10* transcripts were found in oocytes floating in the coelomic cavity and even in yolk-rich eggs. On the other hand, *Plo-piwi1* expression does not last long in oocytes or spermatocytes. In contrast to these three genes, the expression of *Plo-piwiA* and *Plo-piwi2* is not associated with either gonadal development or germ-cell establishment and maintenance.

Similar results were shown for *E. japonensis*. In this oligochaete, transcripts of both *Ej-vlg1* and *Ej-vlg2* (homologs of *Vasa*) were found in the testes, seminal vesicles, and ovaries of mature worms. *Ej-vlg1* was expressed in spermatogonia and spermatocytes, but not in spermatids or in sperms. In contrast, *Ej-vlg2* transcripts were detected in more restricted cells in the seminal vesicle. mRNA of both genes, *Ej-vlg1* and *Ej-vlg2* were observed in oogonia and oocytes in the ovary. *Ej-piwi* expression was also found in the testis, seminal vesicle, and ovary. As in the case of *Ej-vlg1*, *Ej-piwi* mRNAs were detected in spermatogonia and spermatocytes, but not in secondary oocytes [22,23,24]. In *E. japonensis*, it was also found that germ-cell precursors are present in the prospective gonadal region, even in asexually growing animals. After architomy, a kind of fission where an animal splits into fragments before the new head and the new tail develop, gonads can regenerate in each fragment [22].

The emergence of gonad material after such a long period of agamic reproduction raises intriguing questions. Two explanations are theoretically possible. First, we can hypothesize the presence of a specific stem-cell population such as *vasa*- and *piwi*-positive cells in *E. japonensis* [22,23,24]. Indeed, both *P. leidyi* and *P. longiseta* demonstrate the presence of *piwi*-positive cells migrating along the ventral nerve cord ([43,63], and this work). Moreover, in contrast to *P. leidyi*, we found *vasa*-positive cells of similar morphology and localization in *P. longiseta*. This scenario seems to be the most probable, although it does not explain the variability in the presence of such cells. Secondly, it is possible that primordial germ cells form directly from somatic ones by dedifferentiation. Our previous published data suggest a possible migration of dedifferentiated epidermal cells into the segment followed by active cell proliferation which then results in blastemal mass formation in *P. longiseta* [6]. Epidermal cells at the fission zone and in the posterior growth zone showed not only ultrastructural characteristics but also expression patterns of pluripotency markers *vasa*, *pl10*, and *piwi*, very similar to those of blastema cells. Therefore, at least two different scenarios for the origin of the gonad material after a long period of agamic reproduction are possible. Future studies shall involve the identification of evidence for these alternative mechanisms.

## Data Availability

mRNA sequences of *Plo-vasa*, *Plo-pl10*, *Plo-piwiA*, *Plo-piwi2*, and *Plo-piwi1* are deposited in GenBank with the accession numbers JX264563-JX264567, and OR203685.

## Supplementary Materials

The following supporting information can be downloaded at https://www.mdpi.com/article/10.3390/jdb11030034/s1. Table S1. Primer sequences used to clone fragments of the *Plo-vasa*, *Plo-pl10*, *Plo-piwi1*, *Plo-piwiA*, and *Plo-piwi2* genes presented in the paper; Table S2. GenBank access numbers for sequences used for PL10 and VASA amino acid alignments; Table S3. GenBank access numbers for sequences used for PIWI amino-acid alignments; Figure S1. Phylogenetic analysis of *Pristina longiseta Vasa* and *Pl10* homologs. Bayesian consensus tree of the Helicase domains of metazoan *Vasa* and *Pl10* genes; Figure S2. Phylogenetic analysis of *Pristina longiseta Piwi* homologs. Bayesian consensus tree of the Piwi domain of metazoan *Piwi* genes.
