# Supplementary material for: Vasa, Piwi, and Pl10 Expression during Sexual Maturation and Asexual Reproduction in the Annelid Pristina longiseta
Source: J Dev Biol. 2023 Aug 9;11(3):34. doi: 10.3390/jdb11030034 (PMC10443295; doi:10.3390/jdb11030034)

Supplementary Materials

Table S1. Primer sequences used to clone fragments of the *Plo-vasa*, *Plo-pl10*, *Plo-piwi1*, *Plo-piwiA*, and *Plo-piwi2* genes presented in the paper.

<u>*Vasa*, *Pl10*, and *Piwi* short fragments</u>

Vasa, *Pl10*	forward	5’-ATGGCNTGYGCNCARACNGG-3’
	forward nested	5’-CARACNGGNWSNGGNAARACNGC-3’
	reverse	5’-CCNGTNCKNCCDATNCKRTG-3’
	reverse nested	5’-CCDATNCKRTGNACRTAYTC-3’
Piwi	forward	5’-*GGAATTC*TAYCGNGAYGGHGTNGGH-3’
	reverse	5’-CCC*GTCGAC*AAAAGCYAGYTTRTGDGCRTA-3’

<u>*Plo-vasa, Plo-pl10, Plo-piwi1, Plo-piwiA, and Plo-piwi2* RACE fragments</u>

Plo-vasa 3’RACE	forward	5’- GGTCGATTGCTGGACTACATCAAAGAGGG -3’
	forward nested	5’- GCCACCAAAGACCGAACGACAGACAC-3’
Plo-vasa 5’RACE	reverse	5’- CCGACCACGCCAACGACAATGAACAAA -3’
	reverse nested	5’- GTGTCTGTCGTTCGGTCTTTGGTGG -3’
Plo-pl10 3’RACE	forward	5’- CCTGGTCGCCTTGTTGATATGATGGAGAG-3’
	forward nested	5’- CCCCCCACTGGTGATCGTCAAGCCCTG-3’
Plo-pl10 5’RACE	reverse	5’- GAACGCTTGTCTTCTTCCTCCACCC -3’
	reverse nested	5’-CCAACACGGCCAACGGCCAAGAAAAT-3’
Plo-piwi1 3’RACE	forward	5’- CGAGCACTGAAGGAATCTATTGACG -3’
	forward nested	5’- GCCATCAAAACCATCTGCTGTGTTG -3’
Plo-piwi1 5’RACE	reverse	5’- CCCCAACTCCATCACGGAAGACTATG -3’
	reverse nested	5’- GGCATGGTAATTCTTCAAAGCACTCTG-3’
Plo-piwiA 3’RACE	forward	5’- GGAGAAGGACAGCTGAGCACTGTGTATG -3’
	forward nested	5’- CCCAAAGCTGGCGGTGATCGTAGTGTC -3’
Plo-piwiA 5’RACE	reverse	5’- CAGATGGCACAGTTTGTACGTCATTCGC -3’
	reverse nested	5’- GCGTGGGAGTAACGGTTCCTTGTCGG -3’
Plo-piwi2 5’RACE	forward	5’- GGGGCGGGTACACGAACGGTGCCTGG -3’

Table S2. GenBank accession numbers for sequences used for PL10 and VASA amino acid alignments.

**Phylum**	**Species and Gene name in tree**	**GenBank #**
Annelida	(*outgroup*) Tubifex_tubifex_p68_RNA_helicase	BAD90013.1
	Helobdella_robusta_HELRODRAFT_194828	XP_009010523.1
	Pristina_longiseta_PL10	JX264564
	Capitella_teleta_CAPTEDRAFT_224610	ELU14120.1
	Alitta_virens_pl10	AJW77404.1
	Platynereis_dumerilii_PL10b	CAJ15141.1
	Helobdella_robusta_HELRODRAFT_108526	XP_009030586.1
	Capitella_teleta_vasa	DAA06319.1
	Enchytraeus_japonensis_vasa-related_protein	BAF76795.1
	Pristina_leidyi_VASA	AIM52096.1
	Pristina_longiseta_VASA	JX264563
	Alitta_virens_vasa	AJW77403.1
	Platynereis_dumerilii_vasa	CAJ15139.1

Mollusca	Octopus_sinensis_Pl10	XP_029641876.1
	Haliotis_asinina_PL10-like_protein	ACT35658.1
	Pecten_maximus_Pl10	XP_033738811.1
	Crepidula_fornicata_vasa	ADI48178.1
	Azumapecten_farreri_vasa	ABE27759.1
	Crassostrea_gigas_vasa-like_protein	AAR37337.1
Echinodermata	Strongylocentrotus purpuratus_Pl10	XP_030838128.1
	Lytechinus_variegatus_DDX3Y-like	XP_041483221.1
	Strongylocentrotus_purpuratus_DEAD-box_helicase_4	NP_001139665.1
	Lytechinus_variegatus_DED1-like	XP_041475118.1
Chordata	Homo_sapiens_DDX3X	NP_001180345.1
	Mus_musculus_Pl10	NP_149068.1
	Homo_sapiens_DDX4	NP_077726.1
	Mus_musculus_DDX4	NP_001139357.1
	Branchiostoma_floridae_DDX4-like	XP_035672419.1
Cnidaria	Hydra_vulgaris_Pl10	XP_047130892.1

Table S3. GenBank accession numbers for sequences used for PIWI amino acid alignments.

Phylum	Species and Gene name in tree	GenBank #
Annelida	(*outgroup*) Aeolosoma_viride_argonaute_3	AQV09904.1
	Pristina_longiseta_piwi_A	JX264566
	Pristina_longiseta_piwi_1	OR203685
	Pristina_longiseta_piwi_2	JX264567
	Pristina_leidyi_PIWI1	AIM52094.1
	Pristina_leidyi_PIWI2	AIM52095.1
	Capitella_teleta_Piwi1	DAA34898.1
	Capitella_teleta_piwi_2	ELU02261.1
	Aeolosoma_viride_Piwi	AQV09901.1
	Alitta_virens_piwi-like_1	AJW77405.1
	Alitta_virens_piwi-like_2	AJW77406.1
	Platynereis_dumerilii_piwi	CAJ28986.1
	Platynereis_dumerilii_PiwiB	AZG02837.1
	Eisenia_fetida_Piwi2	AUS83924.1
Mollusca	Patella_vulgata_piwi-like_1	XP_050410271.1
	Crassostrea_virginica_piwi-like_1	XP_022338755.1
	Octopus_sinensis_piwi-like_1	XP_029641942.1
Brachiopoda	Lingula_anatina_piwi-like_1	XP_013405373.1
Echinodermata	Strongylocentrotus_purpuratus_seawi	XP_030846820.1
	Lytechinus_variegatus_piwi-like_1	XP_041456323.1
	Patiria_miniata_piwi-like_1	XP_038059144.1
Chordata	Homo_sapiens_piwi-like_1	NP_004755.2
	Mus_musculus_piwi-like_1	NP_067286.1
	Branchiostoma_floridae_piwi-like_1	XP_035691448.1
	Branchiostoma_floridae_Piwi-like_2	AGI95997.1
Cnidaria	Dynamena_pumila_piwi	ULM60680.1
	Podocoryna_carnea_Cniwi	AAS01181.1
	Hydra_vulgaris_PIWI	AFQ20832.1
	Nematostella_vectensis_piwi1	ASW22510.1

Figure S1. Phylogenetic analysis of *Pristina longiseta Vasa* and *Pl10* homologs. Bayesian consensus tree of the Helicase domains of metazoan *Vasa* and *Pl10* genes.

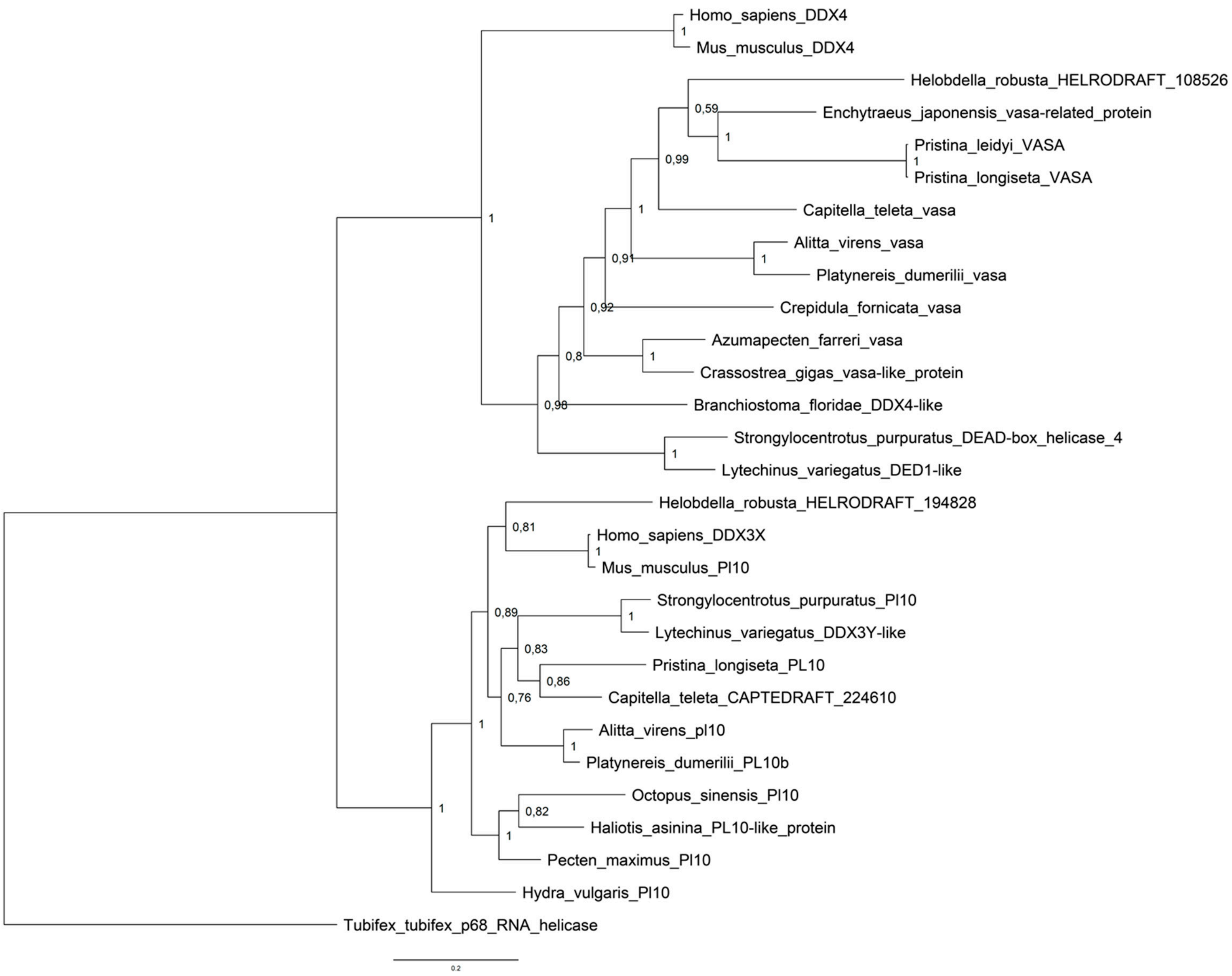

Figure S2. Phylogenetic analysis of *Pristina longiseta Piwi* homologs. Bayesian consensus tree of the Piwi domain of metazoan *Piwi* genes.

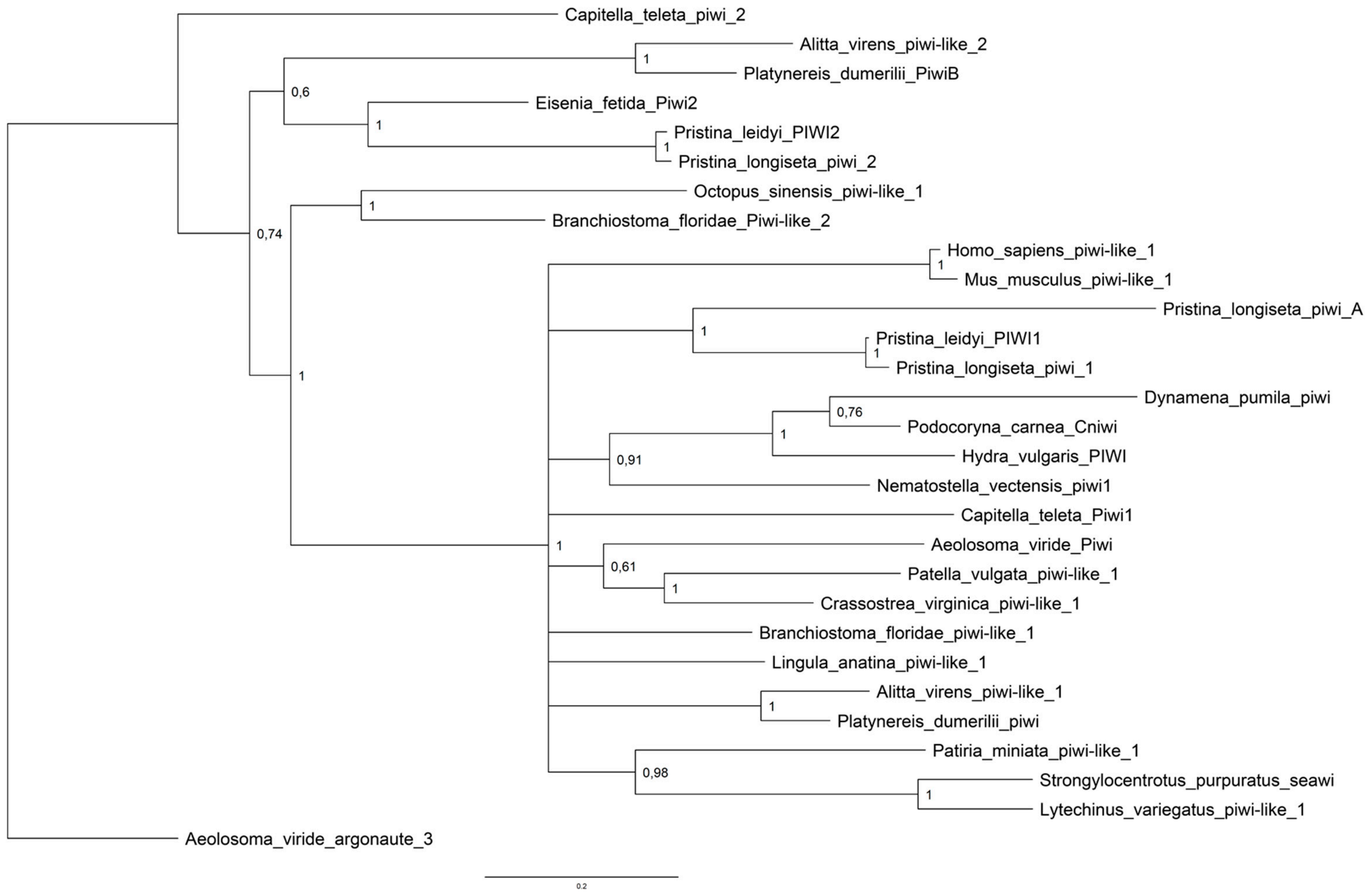

Supplement: Supplementary file 1 [file jdb-11-00034-s001.zip › jdb-2511329-supplementary.pdf]
